# Effect of Cholecalciferol Supplementation on the Clinical Features and Inflammatory Markers in Hospitalized COVID-19 Patients: A Randomized, Open-Label, Single-Center Study

**DOI:** 10.3390/nu14132602

**Published:** 2022-06-23

**Authors:** Tatiana L. Karonova, Ksenia A. Golovatyuk, Igor V. Kudryavtsev, Alena T. Chernikova, Arina A. Mikhaylova, Arthur D. Aquino, Daria I. Lagutina, Ekaterina K. Zaikova, Olga V. Kalinina, Alexey S. Golovkin, William B. Grant, Evgeny V. Shlyakhto

**Affiliations:** 1 Almazov National Medical Research Centre, 197341 Saint Petersburg, Russia; ksgolovatiuk@gmail.com (K.A.G.); igorek1981@yandex.ru (I.V.K.); arabicaa@gmail.com (A.T.C.); armikhaylova@yandex.ru (A.A.M.); akino97@bk.ru (A.D.A.); daria.lagutina.i@yandex.ru (D.I.L.); catherine3452@yandex.ru (E.K.Z.); olgakalinina@mail.ru (O.V.K.); golovkin_a@mail.ru (A.S.G.); shlyakhto_ev@almazovcentre.ru (E.V.S.); 2 Institute of Experimental Medicine, 197376 Saint Petersburg, Russia; 3 Sunlight, Nutrition, and Health Research Center, P.O. Box 641603, San Francisco, CA 94164-1603, USA; williamgrant08@comcast.net

**Keywords:** COVID-19, SARS-CoV-2, vitamin D, 25(OH)D, B cell subsets, inflammatory markers

## Abstract

Recent studies showed that a low 25-hydroxyvitamin D (25(OH)D) level was associated with a higher risk of morbidity and severe course of COVID-19. Our study aimed to evaluate the effects of cholecalciferol supplementation on the clinical features and inflammatory markers in patients with COVID-19. A serum 25(OH)D level was determined in 311 COVID-19 patients. Among them, 129 patients were then randomized into two groups with similar concomitant medication. Group I (*n* = 56) received a bolus of cholecalciferol at a dose of 50,000 IU on the first and the eighth days of hospitalization. Patients from Group II (*n* = 54) did not receive the supplementation. We found significant differences between groups with the preferential increase in serum 25(OH)D level and Δ 25(OH)D in Group I on the ninth day of hospitalization (*p* < 0.001). The serum 25(OH)D level on the ninth day was negatively associated with the number of bed days (r = −0.23, *p* = 0.006); we did not observe other clinical benefits in patients receiving an oral bolus of cholecalciferol. Moreover, in Group I, neutrophil and lymphocyte counts were significantly higher (*p* = 0.04; *p* = 0.02), while the C-reactive protein level was significantly lower on the ninth day of hospitalization (*p* = 0.02). Patients with supplementation of 100,000 IU of cholecalciferol, compared to those without supplementation, showed a decrease in the frequencies of CD38++CD27 transitional and CD27−CD38+ mature naive B cells (*p* = 0.006 and *p* = 0.02) and an increase in the level of CD27−CD38− DN B cells (*p* = 0.02). Thus, the rise in serum 25(OH)D level caused by vitamin D supplementation in vitamin D insufficient and deficient patients may positively affect immune status and hence the course of COVID-19.

## 1. Introduction

Since the beginning of the COVID-19 pandemic, a large amount of data has been accumulated describing the vitamin D status in patients with COVID-19 and its impact on the course and prognosis of a new coronavirus infection. A meta-analysis by Kazemi et al. has illustrated that, in 10 of 12 studies, patients with confirmed COVID-19 had a lower 25(OH)D serum concentration compared to the control group [1].

It was shown that a low 25(OH)D level was associated with a higher risk of morbidity and a severe course of acute respiratory viral infection [2]. It was suggested that a vitamin D deficiency might be one of the modifiable risk factors for new coronavirus infection, worsening the course and prognosis of the disease. Studies over the past two years have found that a low serum 25(OH)D level was associated with the risk of SARS-CoV-2 infection [3] and with the severe course of COVID-19 [4]. Thus, in a study by Mercola et al., patients with a serum 25(OH)D level > 30 ng/mL had mild disease, while patients with a serum 25(OH)D level below 30 ng/mL had a higher severity of COVID-19 and higher mortality rates [4].

Many of the immune cells express vitamin D receptors. Therefore, by its binding, vitamin D can modulate both innate and acquired immune responses [5]. The protective role of vitamin D is linked to several mechanisms: a decrease in neutrophil activity; suppression of the exaggerated activity of type 1 T-helper cells, thus preventing cytokine storm development; and a direct positive effect of the active form of vitamin D on the expression of ACE2, the host receptor for SARS-CoV-2 [6,7,8].

Previous studies have shown that most hospitalized patients with COVID-19 had vitamin D deficiency, which was associated with a 3.79-fold increase in the risk of a severe course of COVID-19 and a 4.07-fold increase in the risk of a fatal outcome. The threshold of a serum 25(OH)D level of 11.4 ng/mL was associated with mortality, as well as with stimulation of type 2 T-helper cells and downregulation of T-helper 17 cell polarization [9,10,11].

Cholecalciferol supplementation, especially in patients with vitamin D insufficiency and deficiency, demonstrated effectiveness in preventing acute respiratory viral infections and COVID-19 [8]. A meta-analysis that included 72 observational and four randomized interventional trials confirmed a correlation between 25(OH)D level and disease severity and mortality. Moreover, clinical benefits, such as a reduction in inflammatory markers, were associated with the addition of vitamin D supplementation to standard COVID-19 therapy [12].

Therefore, we can hypothesize that vitamin D supplementation may be beneficial to reduce morbidity and mortality in COVID-19 patients. Currently, the development of methods for the prevention and treatment of a new coronavirus infection continues. Further studies are needed to evaluate the additive therapeutic efficacy of cholecalciferol in combination with the first-line therapy for COVID-19.

So, this study aimed to evaluate the effects of cholecalciferol supplementation on the clinical features and inflammatory markers in patients with COVID-19.

## 2. Materials and Methods

### 2.1. Patients

We analyzed the vitamin D status of 311 patients hospitalized with COVID-19 (161 men and 150 women). One hundred and twenty-nine patients from 311 patients were randomly included in the interventional study. All patients signed informed consent for participation. The randomized single-center open-label study was performed from 30 November 2020 to 20 March 2021, when the Almazov National Medical Research Centre (St. Petersburg, Russia) was transformed into an infectious hospital for COVID-19 patients. The local Ethics Committee of the Almazov National Medical Research Centre approved this study (protocol No. 1011-20-02C, 30 November 2020), which complied with the Declaration of Helsinki. This study was registered on clinicaltrials.gov (NCT number: NCT05166005).

The inclusion criteria included: age from 18 to 75 years, confirmed diagnosis of COVID-19 (polymerase chain reaction (PCR)-test and/or chest computed tomography (CT) scan), and signed informed consent. Subjects with daily vitamin D intake of 1000 IU and higher or who had contraindications to vitamin D supplementation were not included. Additional exclusion criteria were clinically significant kidney pathology with an eGFR of less than 45 mL/min/1.73 m^2^; gastrointestinal and liver diseases; granulomatous diseases; oncology diseases (less than 5 years); immunodeficiency disorders; and addiction to drugs and alcohol. We did not include pregnant or breastfeeding women. Potential subjects with other circumstances considered inappropriate by the investigator were not allowed to participate in the study. All participants were unvaccinated, since general vaccination was not yet available.

One hundred and twenty-nine patients were randomized by random number table into two groups depending on vitamin D supplementation (water-soluble cholecalciferol): Group I received a bolus of cholecalciferol at a dose of 50,000 IU on the 1st and the 8th day of hospitalization, with the total dose being 100,000 IU; Group II received no supplementation.

### 2.2. Clinical Data

We analyzed the following clinical data: height, weight, body mass index (BMI), and co-morbidities. We assessed the severity of the disease by oxygen supplementation, SpO2, the time between symptom onset and hospitalization, intensive care unit admission rates, and bed days. The disease severity was classified according to the following criteria: mild illness—temperature < 38 °C, absence of shortness of breath, dyspnea or normal chest computed tomography (CT); moderate illness—temperature > 38 °C, SpO2 < 95%, C-reactive protein (CRP) > 10 mg/L, CT—1 or 2; and severe illness—hemodynamic instability, SpO2 < 93%, CT—3 or 4 [13].

### 2.3. Laboratory Tests

Laboratory parameters of serum 25(OH)D level, complete blood count, and the acute phase proteins, including CRP, lactate dehydrogenase (LDH), and ferritin, were measured at baseline. On the 9th day of vitamin D supplementation, we assessed serum 25(OH)D level, complete blood count, and CRP level.

We measured serum 25(OH)D levels using a chemiluminescence immunoassay on microparticles (Abbott Architect i8000, Chicago, IL, USA); the reference interval was 3.4–155.9 ng/mL, intra-assay coefficient of variation ranged from 1.60% to 5.92%, and inter-assay coefficient of variation ranged from 2.15% to 2.63%. Blood samples for 25(OH)D measurements were taken in the morning from the cubital vein, centrifuged, aliquoted, and stored in a freezer at a temperature of −70 °C before the laboratory testing. According to a current vitamin D supplementation guideline, vitamin D status was considered normal when the 25(OH)D level was ≥30 ng/mL (≥75 nmol/L); for insufficiency, the 25(OH)D level was ≥20 and <30 ng/mL (≥50 and <75 nmol/L); for deficiency, the 25(OH)D level was <20 ng/mL (<50 nmol/L) [14].

We used a Cobas Integra 400analyzer (Roche Diagnostics GmbH, Mannheim, Germany) and corresponding diagnostic kits to determine the CRP level (reference range 0–5 mg/L) and LDH level (reference range 133–225 units/L). Ferritin level was measured on an Abbott Architect c8000 analyzer (Chicago, IL, USA; reference range, 64–111 nmol/L).

### 2.4. Instrumental Data

To detect pneumonia, we used CT scans without intravenous contrast enhancement. The volume of lung tissue lesions was described as CT-1, lesion volume < 25%; CT-2, lesion volume 25–50%; CT-3, lesion volume 50–75%; and CT-4, lesion volume > 75% [13].

### 2.5. Concomitant Medication

Concomitant medication for COVID-19 was analyzed for each patient group. Treatment was carried out according to the local guidelines [13]. The analysis included evaluation of anti-IL-6 receptor monoclonal antibodies and glucocorticoids (GC) administration, as well as calculation of total GC dose from the 1st until the 9th day.

### 2.6. Immunological Data

The frequencies of peripheral blood B cell subsets in patients with COVID-19 were analyzed by flow cytometry. Staining protocol, reagents, and gating strategy were described in detail earlier [15]. We classified B cell subsets using CD27 and CD38 co-expression, as was suggested by P. Hanley et al. [16]. As a result, we identified six main B cell subsets, including CD27−CD38++ transitional B cells, CD27++CD38++ circulating plasma cell precursors, mature naïve and mature activated B cells (CD27−CD38+ and CD27+CD38+, respectively), CD27−CD38− double-negative or DN B cells and, finally, CD27+CD38− resting memory B cells.

### 2.7. Study Objective

The primary outcomes were changes in serum 25(OH)D level, complete blood count, CRP level in peripheral blood, and B cell subsets on the 9th day of hospitalization compared to the first day. The secondary endpoint was to evaluate the effects of cholecalciferol supplementation on the severity of the disease, oxygen supplementation, intensive care unit admission rates, and clinical outcomes. The additional secondary endpoint was the duration of hospitalization.

### 2.8. Statistical Analysis

For sample size calculation, we used Power and Sample Size software (Sealedenvelope, 2022; London, UK). At the 95% confidence level, 80% power, and 10% estimated dropout rate from the study protocol after randomization, the optimal sample size was determined as 118 participants (59 per group).

Statistical processing was conducted using Jamovi Software, version 2.3.2 (Jamovi project, 2022; Sydney, Australia). Results are presented as the median (Me) and interquartile range [25%; 75%]. A Mann–Whitney U-test was carried out to compare the means of the two groups. The statistically significant differences between the Me [25%; 75%] on the 1st day and on the 9th day of hospitalization were assessed using the Wilcoxon W test. Spearman’s correlation coefficient was used for associations between quantitative parameters. A *p*-value of <0.05 was the criterion for the statistical reliability of the obtained results.

## 3. Results

Vitamin D status was determined in 311 patients with confirmed COVID-19. Sixty-nine patients (22.2%) had a normal vitamin D status, 57 (18.3%) had an insufficiency, and 185 (59.5%) had a deficiency. The serum 25(OH)D levels were measured simultaneously in all stored samples after the interventional study.

**Figure 1:**
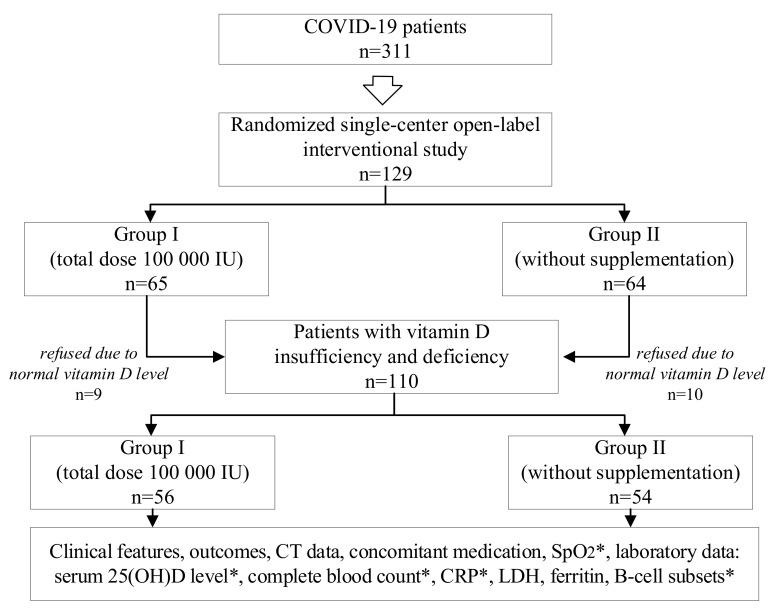
Study design. CT, computed tomography; SpO2, oxygen saturation; 25(OH)D, 25-hydroxyvitamin D; CRP, C-reactive protein; LDH, lactate dehydrogenase; *, both on the 1st and at the 9th day.

One hundred and twenty-nine COVID-19 patients were randomly chosen from 311 and included in the interventional study: Group I (*n* = 65) and Group II (*n* = 64) (Figure 1). The study design is illustrated in Figure 1.

Patients’ baseline characteristics are presented in Table 1.

The groups were comparable and had no significant differences in baseline parameters, including serum 25(OH)D level, and clinical course of the disease, including CT data and oxygenation parameters (*p* > 0.05). Patients in Group I were significantly younger than patients in Group II (*p* = 0.03).

Participants in Group I had a normal serum 25(OH)D level in nine cases (13.8%), 20 patients (30.8%) had an insufficient level, and 36 (55.4%) had a deficiency. In Group II, 10 patients (15.6%) had a normal level, 11 (17.2%) patients had an insufficiency, and 43 (67.2%) had a deficiency. Thus, 19 (14.7%) of the 129 participants had a normal serum 25(OH)D level on the 1st day of hospital admission. Therefore, their data were not considered for further analysis of the cholecalciferol supplementation effect on the clinical and laboratory parameters (Table 2).

After the exclusion of subjects with a normal baseline 25(OH)D level, the groups remained comparable and had no significant differences in baseline parameters. There were no significant differences in serum 25(OH)D levels between the groups (*p* = 0.08). The analysis of the concomitant medication showed the absence of significant differences in the groups (*p* > 0.05). However, patients in Group I were still younger than patients in Group II (*p* = 0.03).

After the initiation of vitamin D supplementation, we performed a comparative analysis between the groups to assess the parameters on the 9th day of hospitalization (Table 3).

In Group I (*n* = 56), on the 9th day after 100,000 IU cholecalciferol supplementation, the median serum 25(OH)D level was 22.8 ng/mL [17.7;27.7]. The absolute and the relative Δ 25(OH)D were 6.2 ng/mL [2.4;11] and 40.7% [14.0;78.4], respectively. At the same time, in Group II (*n* = 54), the median level of 25(OH)D on the 9th day was 10.6 ng/mL [8.4;14.9]. The absolute and the relative Δ 25(OH)D were 2.6 ng/mL [−4.3;0] and −18.2% [−28.8;0], respectively.

**Figure 2:**
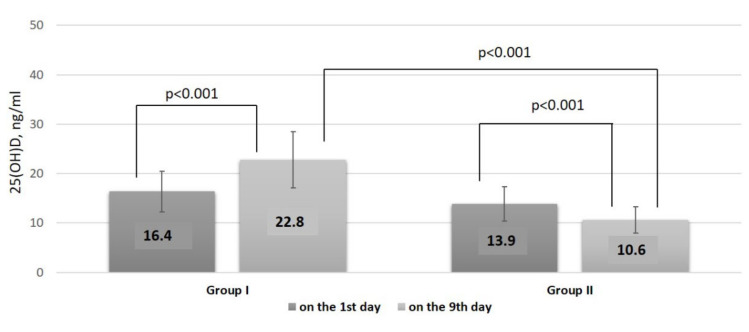
Serum 25(OH)D level before and after supplementation with 100,000 IU of cholecalciferol.

Thus, we found significant differences on the 9th day of hospitalization between the groups in vitamin D status, serum 25(OH)D level, and Δ 25(OH)D (*p* < 0.001) (Figure 2). Furthermore, the serum 25(OH)D level on the 9th day was negatively associated with the number of bed days (r = −0.23, *p* = 0.006).

**Figure 3:**
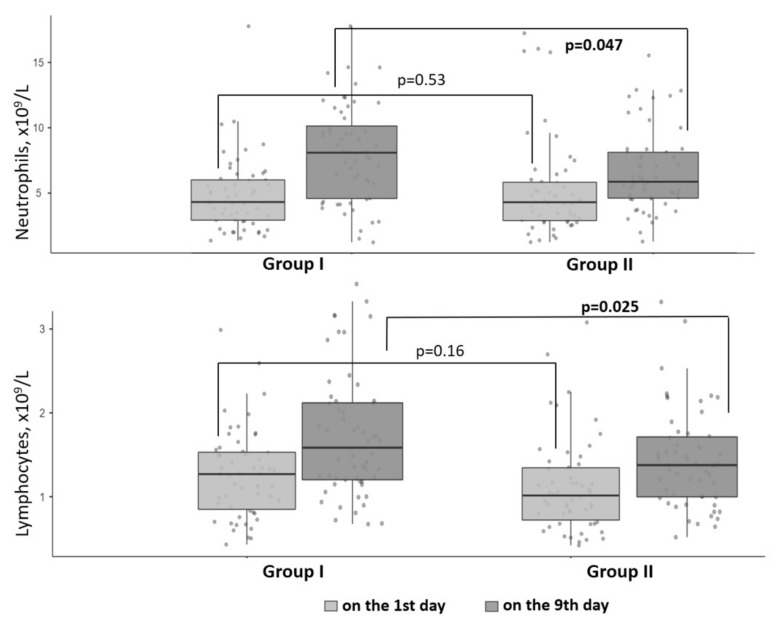
Neutrophil and lymphocyte counts before and after 100,000 IU cholecalciferol supplementation.

When comparing the results of the complete blood count, neutrophil and lymphocyte counts were significantly higher in Group I (*p* = 0.04; *p* = 0.02) (Figure 3).

Additionally, the CRP level on the 9th day of hospitalization was significantly lower among patients in Group I (*p* = 0.02). There was also a negative association between CRP and serum 25(OH)D level (r = −0.28, *p* = 0.02).

**Figure 4:**
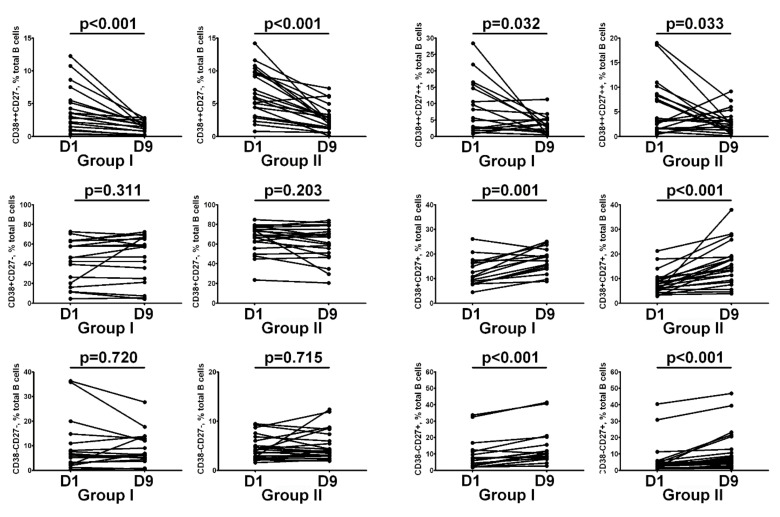
Main B cell subsets frequencies before and after 100,000 IU cholecalciferol supplementation. Numbers represent the percentages of the indicated B cell subset among the total B cell population. Each pair of connected points represents an individual subject. Day 1 vs. day 9 post-hospitalization intra-individual patient samples were compared by Wilcoxon matched-pairs signed rank test with two-tailed *p* value.

We next addressed whether the circulating B cell subsets were stable in groups I and II or changed between the 1st and 9th days of hospitalization. We also observed that the subpopulation of CD38++CD27 transitional B cells was decreased, while mature activated B cells (CD27+CD38+) and resting memory B cells (CD27+CD38−) frequencies increased in most subjects from both groups of COVID-19 patients (Figure 4). Furthermore, the relative numbers of mature naive CD27−CD38+ B cells, circulating plasmablast precursors CD27++CD38++, and double-negative CD27−CD38− B cells did not change significantly over time.

**Figure 5:**
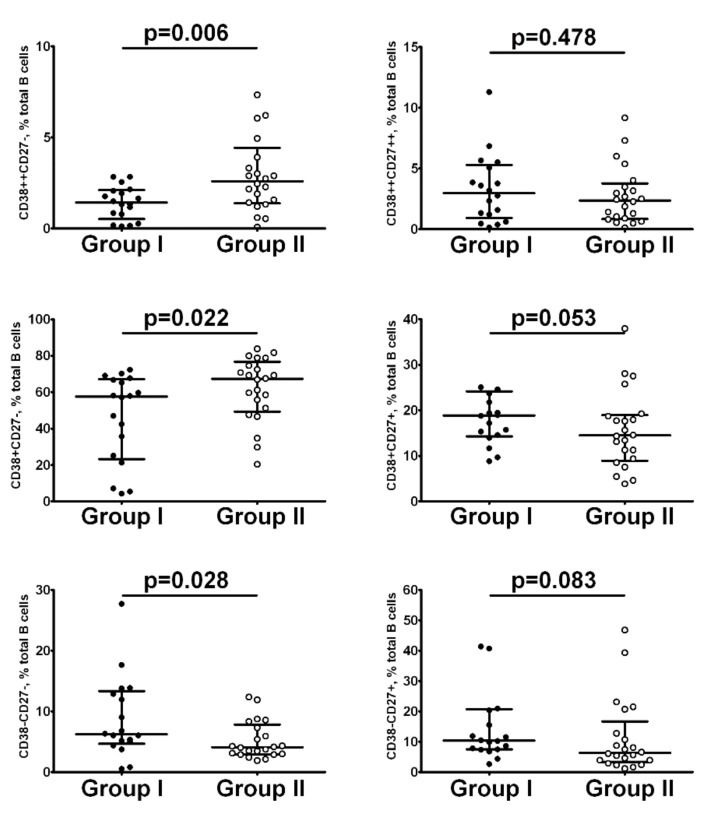
The impact of 100,000 IU cholecalciferol supplementation on B cell subset frequencies on the 9th day of hospitalization. Black circles—patients from Group I (*n* = 18); open circles—patients from Group II (*n* = 22). Numbers represent the percentages of the indicated B cell subset among the total B cell population. Each dot represents individual subject. Horizontal bars depict the group medians and interquartile ranges (Med (Q25; Q75)). Statistical analysis was performed with the Mann–Whitney U test.

Finally, we compared the relative numbers of B cell subsets in Groups I and II on day 9 post-hospitalization. We found that after 100,000 IU cholecalciferol supplementation, patients with COVID-19 had decreased frequencies of CD38++CD27 transitional and mature naive CD27−CD38+ B cells if compared to Group II (1.43% (0.79; 2.08) vs. 2.74% (1.43; 3.91), *p* = 0.006 and 57.57% (25.15; 66.82) vs. 67.03% (51.16; 74.71), *p* = 0.02, respectively). We also noticed that the level of CD27−CD38− DN B cells was increased in patients from Group I when compared to Group II patients (6.21% (4.96; 12.91) vs. 4.19% (3.04; 7.33), *p* = 0.02) (Figure 5).

## 4. Discussion

Recently published systematic reviews and meta-analyses have demonstrated that vitamin D insufficiency and deficiency are highly prevalent in patients with moderate and severe COVID-19 [1,17]. At present, there are sufficient data to demonstrate that a low serum 25(OH)D concentration increases the disease severity and risk of death in patients with COVID-19 [10,18]. A study conducted in the United States showed that patients with a positive SARS-CoV-2 test and a 25(OH)D concentration of 15 ng/mL compared to 40 ng/mL had a 20% greater risk of hospitalization (*p* = 0.009) and an increased risk of mortality by 53% (*p* = 0.001) [19]. The same results were obtained in a study of 311 hospitalized COVID-19 patients: low serum 25(OH)D concentrations were found in patients with poorer clinical outcomes compared to those with a moderate and mild clinical course (*p* = 0.001) [10].

There is no definitive position regarding the additive therapeutic efficacy of vitamin D combined with standard treatment. Many studies showed a positive effect of vitamin D supplementation on the course and prognosis of COVID-19 [20,21,22,23,24]. In the observational study of Ling et al., cholecalciferol treatment using high-dose booster therapy (approximately ≥ 280,000 IU over a period of up to 7 weeks) was associated with a reduced risk of COVID-19 mortality in the cohort of 444 patients [25]. Torres et al. demonstrated that a daily dose of 10,000 IU of cholecalciferol increased serum 25(OH)D levels to 29 ng/mL on average vs. 19 ng/mL in the group receiving 2000 IU/day, after 7 and 14 days of treatment (*p* < 0.0001). The beneficial effect of supplementation with 10,000 IU/day was observed in participants with COVID-19 and acute respiratory distress syndrome who stayed in the hospital for 8 days. In contrast, those who received 2000 IU/day stayed for 29 days (*p* = 0.03) [26]. Another study using cholecalciferol at doses ranging from 224,000 to 500,000 IU over 3–14 days, in 132 COVID-19 patients with baseline serum 25(OH)D level of <30 ng/mL, showed a significant decrease in 14-day mortality (OR for survival: 2.14, 95% CI: 1.06 to 4.33, *p* = 0.03), when 25(OH)D levels of 31 ± 12 ng/mL on the 7th day and 35 ± 11 ng/mL on the 14th day were achieved [27].

A study from Spain included 527 patients with COVID-19; among them, 79 patients received calcifediol treatment (532 μg on entry and then 266 μg on days 3, 7, 14, 21, and 28). The calcifediol treatment was associated with significantly lower in-hospital mortality during the first 30 days [28]. The results of a large population-based study that compared patients receiving cholecalciferol or calcifediol (>250 μg of cholecalciferol or calcifediol as a bolus dose) showed that achieving serum 25(OH)D levels of ≥30 ng/mL improved the clinical outcomes of COVID-19 [29].

Calcifediol had better effects on COVID-19 outcome due to its ability to rapidly increase serum 25(OH)D levels compared to cholecalciferol. For example, the Gonen trial study found very little effect with high-dose vitamin D3 [27], while the Spanish studies generally demonstrate high beneficial effects for calcifediol [28]. The rapid response to treatment is of great importance since the main therapeutic effects of vitamin D are linked to the reduction in the viability and replication of SARS-CoV-2 and to the down-regulation of the production of the pro-inflammatory cytokines, thus decreasing the risk of the cytokine storm that causes organ damage [28,29].

We initiated this study assuming that vitamin D supplementation in hospitalized subjects might improve clinical outcomes and decrease inflammatory markers. At the end of the study, we showed that the use of 100,000 IU cholecalciferol supplementation in addition to standard COVID-19 therapy led to an increase in serum 25(OH)D levels by 40.7%, thus preventing vitamin D deficiency in the acute period of COVID-19. On the other hand, we have found no differences in mortality, ICU admission rates, and the average time of hospital stay between the supplemented and control groups. However, these results may be related to the fact that we did not achieve the recommended 25(OH)D level of 40 to 60 ng/mL. Similar results were obtained in other studies; there were no statistically significant differences between the groups receiving only standard therapy and the groups supplemented with vitamin D [24,30]. For example, in a study of 240 hospitalized patients with moderate and severe COVID-19, a single dose of vitamin D supplementation (200,000 IU) did not significantly reduce bed days, ICU admission rates, or mortality. These conflicting findings can probably be explained by the different doses and frequencies of administration, size samples, and principle of the sampling of patients included in the studies.

Even before the pandemic, there was evidences that GC therapy was associated with a decrease in serum 25(OH)D levels [31]. This is also a common phenomenon in patients with moderate and severe COVID-19. The results of the present study partially support these findings, confirming a negative dynamic of serum 25(OH)D levels in patients who received GC therapy without vitamin D supplementation.

The severity of COVID-19 is determined by the hyperactive immune response and cytokine storm, accompanied by dysregulated innate and adaptive immune responses [15,32]. Previous studies showed that COVID-19 patients deficient in vitamin D demonstrated higher levels of acute phase reactants compared to those with a normal serum 25(OH)D concentration [10,21]. Rastogi et al. showed a significant decrease in fibrinogen levels after cholecalciferol supplementation at a dose of 60,000 IU daily, with a therapeutic target of 25(OH)D > 50 ng/mL [22].

High serum CRP levels are key markers of disease progression and a risk factor for mortality in patients with severe COVID-19, and indicate the development of a cytokine storm. In the present study, patients receiving cholecalciferol therapy had a significant decrease in CRP level compared to patients without supplementation on the 9th day of hospitalization (*p* = 0.02). In addition, a negative correlation was found between the serum 25(OH)D level and CRP level on the 9th day of hospitalization (r = −0.28, *p* = 0.02).

Viral infections lead to dynamic changes in the peripheral blood leukocyte count and its subsets. A sustained decrease in the peripheral blood lymphocyte count is considered as an early indicator of severe/critically ill patients with COVID-19 [33]. Our previous study showed that vitamin D deficiency was associated with a greater decrease in absolute lymphocyte count [11]. When we compared complete blood counts on the 9th day of hospitalization, the group receiving a vitamin D bolus dose had higher levels of neutrophils and leukocytes (*p* = 0.04; *p* = 0.02). Similar results were previously described in the randomized, placebo-controlled clinical trial. Treatment with oral vitamin D resulted in a significant increase in the lymphocyte percentage and a decrease in the neutrophil-to-lymphocyte ratio in the patients [34].

Different types of innate and adaptive immune cells, including antigen-presenting dendritic cells, tissue-resident macrophages, and peripheral blood circulating monocytes, as well as T- and B-lymphocytes, express the vitamin D receptor and are able to modify their functional activity in response to vitamin D stimulation [35,36]. Currently, within adaptive immune responses, the effects of vitamin D have been studied well for T cells and their separate subsets, including CD4+ T cells, CD8+ T cells, and regulatory T cells [37,38]. However, the data on the influence of vitamin D on B cell functions are scarce.

Previously it was found that vitamin D receptor signaling could reduce or even prevent activation of B cells due to modulation of NF-κB mediated activation of naïve B cells [39] Moreover, vitamin D inhibited the proliferation of activated B cells, induced their apoptosis, and down-regulated generation of plasma cells and post-switch memory B cells [40]. Finally, vitamin D receptor activation induced in vitro production of anti-inflammatory IL-10 by B cells [41]. Thus, vitamin D deficiency could dramatically influence B cell functions and negatively affect humoral immune responses [42]. Interestingly, low levels of vitamin D have been closely linked with increased morbidity in several infectious diseases [43]. Oppositely, increased levels of vitamin D showed an inverse association with the development of several autoimmune diseases, such as systemic lupus erythematosus, thyrotoxicosis, multiply sclerosis, iridocyclitis, Crohn’s disease, ulcerative colitis, psoriasis vulgaris etc., pointing to its anti-inflammatory role during immune responses [44]. Furthermore, during acute SARS-CoV-2 infection, vitamin D, in addition to standard therapies, significantly reduced several inflammation markers including the concentrations of C-reactive protein, IL-6, ferritin, and the neutrophil-to-lymphocyte ratio [45], as well as faster improvement in some clinical symptoms [46] and the SaO2/FiO2 ratio [47].

Previously, it was noted that almost all innate and adaptive immune cell subsets were altered during acute COVID-19 [48,49,50]. Furthermore, it was noted that key alterations in peripheral blood circulating B cell populations during the acute phase of SARS-CoV-2 infection were linked to the decreased levels of the relative and absolute number of B cells, increased frequencies of plasma cell precursors, atypical CD21-negative and activated B cells etc. [49,51,52]. Currently, we have shown that vitamin D supplementation could effectively decrease the relative numbers of activated CD38++CD27 transitional B cells and ‘naïve’ B cells in patients with acute COVID-19. Similarly, in systemic lupus erythematosus, vitamin D down-regulated B cell activity in vivo and in vitro [40,53,54]. Thus, altered B cell activation in circulation and vitamin D deficiency could be associated with an increased risk of autoimmune disorders and poor outcomes of infectious diseases. Finally, an increased level of activated B cells and altered B cell subset composition was detected in the peripheral blood of patients recovered after COVID-19 [55,56]; thus, future studies will be needed to investigate whether supplementation with regular vitamin D can prevent or reduce the risk of developing severe pathologies associated with ‘post-COVID’ syndrome.

To sum up, most hospitalized patients with moderate and severe COVID-19 had vitamin D deficiency or insufficiency. An increase in the serum 25(OH)D level may positively affect the course of COVID-19 and patient laboratory parameters. We demonstrated that bolus doses of cholecalciferol resulted in an increased serum 25(OH)D level, neutrophil and lymphocyte counts, and decreased CRP levels in the acute period of COVID-19. In addition, intake of 100,000 IU cholecalciferol was associated with a decline in CD38++CD27 transitional and CD27−CD38+ mature naive B cells and a rise in CD27−CD38− DN B cells. The present study corroborates the therapeutic efficacy of cholecalciferol as a supplement to standard therapy for COVID-19.

## 5. Limitations

Possible study limitations include the small sample size and application of small doses of vitamin D, thus the inability to reach the target 25(OH)D level. Moreover, in the present study, the treatment group was slightly younger than the control group; although, at the same time both groups did not differ in other assessed parameters. In addition, the study treatment design was open-label.

## Figures and Tables

**Table 1:** Patients’ baseline characteristics (*n* = 129).

Parameters	Group I *n* = 65	Group II *n* = 64	*p*
Age, years, Me and IQR [25; 75]	57 [51; 66]	64 [55; 70]	0.03
Gender, female, *n* (%)	31 (47.7)	32 (50.0)	0.86
Days from symptoms onset to hospitalization, days, Me and IQR [25; 75]	8 [6;10]	8 [6;10]	0.37
Severe clinical course, *n* (%)	13 (20)	13 (20)	0.36
CT lung involvement, %, Me and IQR [25; 75]	39 [30; 50]	30 [20; 45]	0.06
CT grading, *n* (%)			*0.29*
*0*	*4 (6)*	*2 (3)*	
*1*	*10 (15)*	*20 (30)*	
*2*	*37 (57)*	*33 (52)*	
*3*	*12 (17)*	*6 (10)*	
*4*	*2 (3)*	*3 (5)*	
SpO2, %, Me and IQR [25; 75]	95 [92; 97]	95 [92; 97]	0.51
Supplemental Oxygenation, *n* (%)	38 (58.4)	32 (50)	0.35
BMI, kg/m^2^, Me and IQR [25; 75]	29.5 [25.5; 32.9]	28.9 [25.5; 31.4]	0.41
Obesity, *n* (%)	28 (43.1)	22 (34.9)	0.42
DM type 2, *n* (%)	17 (26.2)	24 (38.1)	0.84
AH, *n* (%)	46 (70.8)	49 (76.6)	0.31
IHD, *n* (%)	16 (24.6)	14 (21.9)	0.12
Neutrophils, ×10^9^/L, Me and IQR [25; 75]	4.5 [2.4; 7.1]	4.2 [2.9; 5.9]	0.80
Lymphocytes, ×10^9^/L, Me and IQR [25; 75]	1.3 [0.8; 1.5]	1.04 [0.7; 1.4]	0.25
NLR, Me and IQR [25; 75]	3.7 [2.5; 7.6]	4.3 [2.7; 8]	0.15
CRP, mg/L, Me and IQR [25; 75]	48 [21; 134]	49 [18; 107]	0.73
Ferritin, ng/mL, Me and IQR [25; 75]	610 [243; 610]	446.1 [237; 825]	0.12
LDH, µ/L, Me and IQR [25; 75]	351 [261; 483]	327.5 [265; 495]	0.80
25(OH)D, ng/mL, Me and IQR [25; 75]	17.8 [11.7; 25.4]	15.4 [11.0; 22.9]	0.47
Vitamin D status, *n* (%)			0.07
*Normal*	*9 (13.8)*	*10 (15.6)*	
*Insufficiency*	*20 (30.8)*	*11 (17.2)*	
*Deficiency*	*36 (55.4)*	*43 (67.2)*	

CT, computed tomography; BMI, body mass index; DM, diabetes mellitus; IHD, ischemic heart disease; AH, arterial hypertension; NLR, neutrophil/lymphocyte ratio; CRP, C-reactive protein; LDH, lactate dehydrogenase; Me, median; IQR, interquartile range.

**Table 2:** Patients’ baseline characteristics with vitamin D insufficiency and deficiency (*n* = 110).

Parameters	Group I *n* = 56	Group II *n* = 54	*p*
Age, years, Me and IQR [25; 75]	58 [50; 65]	64 [55; 70]	0.03
25(OH)D, ng/mL, Me and IQR [25; 75]	16.4 [11.0; 21.8]	13.9 [9.7; 17.4]	0.08
Vitamin D status, *n* (%)			0.07
*Insufficiency*	20 (36)	11 (20)	
*Deficiency*	36 (64)	43 (80)	
CT lung involvement, %, Me and IQR [25; 75]	42 [30; 48.5]	32.5 [20.5; 45]	0.21
CT grading, *n* (%)			0.77
*1*	11 (19.6)	19 (35.2)	
*2*	33 (58.9)	26 (48.1)	
*3*	11 (19.6)	6 (11.1)	
*4*	1 (1.9)	3 (5.6)	
SpO2, %, Me and IQR [25; 75]	95 [92; 97]	95 [92; 97]	0.50
Supplemental Oxygenation, *n* (%)	38 (68)	32 (59)	0.35
Neutrophils, ×10^9^/L, Me and IQR [25; 75]	4.3 [2.9; 6.0]	4.3 [2.9; 5.8]	0.53
Lymphocytes, ×10^9^/L, Me and IQR [25; 75]	1.3 [0.9; 1.5]	1.0 [0.7; 1.3]	0.16
NLR, Me + IQR [25; 75]	3.5 [2.2; 5.3]	4.7 [2.6; 7.3]	0.09
CRP, mg/L, Me and IQR [25; 75]	48.2 [22.7; 135.3]	47.5 [17.5; 99.0]	0.97
Ferritin, ng/mL, Me and IQR [25; 75]	559 [217; 925]	365 [229; 765]	0.21
LDH, µ/L, Me and IQR [25; 75]	351 [261; 516]	327 [261; 496]	0.84
Concomitant medication			
*GC*			
*Dexamethasone, n (%)*	*47 (84)*	*43 (80)*	*0.67*
*Dexamethasone, mg*	*136 [72; 214]*	*149 [112; 234]*	*0.99*
*Prednisolone, n (%)*	*15 (26.7)*	*11 (20.3)*	*0.53*
*Prednisolone, mg*	*1295 [846; 1658]*	*1140 [375; 1703]*	*0.60*
Anti-IL-6 receptor monoclonal antibodies, *n* (%)	16 (28.5)	18 (33.3)	0.59
*Olokizumab, n (%)*	*13 (23.2)*	*15 (27.7)*	*0.39*
*Levilimab, n (%)*	*2 (3.5)*	*2 (3.7)*	*0.38*
*Tocilizumab, n (%)*	*4 (7.14)*	*3 (5.55)*	*0.74*
Anticoagulant therapy, *n* (%)	56 (100)	54 (100)	-
Antibiotics therapy, *n* (%)	8 (12.5)	11 (20.4)	0.26

25(OH)D, 25-hydroxyvitamin D; SpO2, oxygen saturation; NLR, neutrophil/lymphocyte ratio; CRP, C-reactive protein; LDH, lactate dehydrogenase; Me, median; IQR, interquartile range.

**Table 3:** Patients’ characteristics on the 9th day of hospitalization (*n* = 110).

Parameters	Group I *n* = 56	Group II *n* = 54	*p*
Vitamin D status, *n* (%)			
*Normal*	13 (23)	1 (2)	
*Insufficiency*	20 (36)	3 (6)	
*Deficiency*	23 (41)	50 (92)	<0.001
25(OH)D, ng/mL, Me and IQR [25; 75]	22.8 [17.7; 27.7]	10.6 [8.4; 14.9]	<0.001
Bed days, Me and IQR [25; 75]	18 [14; 22]	17 [14; 23]	0.87
Discharged, *n* (%)	56 (100)	54 (100)	0.93
ICU admission rates, *n* (%)	0	3 (6)	-
SpO2, %, Me and IQR [25; 75]	97 [96; 98]	97 [96; 98]	0.56
Supplemental Oxygenation, *n* (%)	27 (48)	28 (52)	0.70
Neutrophils, ×10^9^/L, Me and IQR [25; 75]	8.6 [5.1; 10.6]	6.4 [5.2; 8.6]	0.04
Lymphocytes, ×10^9^/L, Me and IQR [25; 75]	1.8 [1.3; 2.6]	1.58 [1.0; 2.0]	0.02
NLR, Me and IQR [25; 75]	4.5 [2.6; 6.9]	4.4 [2.7; 7.0]	0.71
CRP, mg/L, Me and IQR [25; 75]	2 [0.8; 4.7]	3 [1; 9]	0.02

25(OH)D, 25-hydroxyvitamin D; ICU, intensive care unit; SpO2, oxygen saturation; NLR, neutrophil/lymphocyte ratio; CRP, C-reactive protein; LDH, lactate dehydrogenase; Me, median; IQR, interquartile range.

## Data Availability

The data generated and analyzed during this study are included in this published article. Additional information is available from the corresponding author on reasonable request.
